# Experimental Investigation of the TRM-to-Masonry Bond after Exposure to Elevated Temperatures: Cementitious and Alkali-Activated Matrices of Various Densities

**DOI:** 10.3390/ma15010140

**Published:** 2021-12-25

**Authors:** Paraskevi D. Askouni, Catherine (Corina) G. Papanicolaou, Lazar Azdejkovic

**Affiliations:** Department of Civil Engineering, University of Patras, Rio, 26504 Patras, Greece; kpapanic@upatras.gr (C.G.P.); lazar@upatras.gr (L.A.)

**Keywords:** textile reinforced mortar (TRM), masonry, temperature, cementitious/alkali-activated matrix, normal-weigh/lightweight aggregates

## Abstract

Limited research has focused on the effect of high temperatures on the textile-reinforced mortar (TRM)-to-masonry bond. In this study, masonry prisms that were furnished with double-layered TRM strips were tested under shear bond conditions after their exposure to 200 °C and 400 °C for 1 h using the single-lap/single-prism setup. A total of four TRM systems were applied sharing the same type of textile –a dry AR glass fiber one– and different matrices: two cementitious matrices, namely a normal-weight (TRCNM) and a lightweight (TRCLM) one, and two counterpart alkali-activated matrices (TRAANM and TRAALM) based on metakaolin and fly ash. Specimens’ exposure to elevated temperatures did not alter their failure mode which was due to the sleeve fibers’ rupture along with core fibers’ slippage from the mortar. The residual bond capacity of the TRM systems decreases almost linearly with increasing exposure temperature. The alkali-activated textile reinforced mortars outperformed their cement-based counterparts in terms of bond strength at every temperature. All systems retained close to 50% of their original shear bond strength after heating at 400 °C. Per the type of binder, lightweight matrices resulted in either comparable (cement-based systems) or better (alkali-activated systems) heat protection at the TRM/masonry interface.

## 1. Introduction

Textile reinforced mortar (TRM) is an innovative composite material that has been developed over the last 15 years and is suitable for the strengthening of existing structures. It consists of a fibrous mesh that is sandwiched between layers of an inorganic matrix and is applied as an external reinforcement on masonry or concrete substrates in the form of single (one textile) or multiple (up to four textiles) overlays. A wide variety of textiles is offered by industries in terms of geometry (2d grids with fiber rovings that are arranged at two orthogonal directions and/or at ±45° or 3d grids), type of fibers (carbon, glass, basalt, aramid, PBO, flax, etc.), and rovings’ treatment (polymer coating or impregnation, dry fibers). In addition, many types of mortars can be applied as a matrix such as cement-, lime-, and gypsum-based as well as alkali-activated ones.

The TRM strengthening technique has already been adopted for both masonry and concrete structure rehabilitation schemes, [1]. This composite material is an interesting strengthening option, especially for masonry structures, since it is characterized by a high strength-to-weight ratio, offers vapor permeability of the substrate, and can be applied under low temperature and/or high humidity conditions. The reversibility of TRM and its compatibility with various substrate materials through the use of appropriate matrices render this technique an attractive strengthening solution for monumental structures as well.

Due to its inorganic matrix, TRM is incombustible. Nevertheless, its actual response during or after exposure to elevated/high temperatures or fire has not yet been largely investigated. The relevant literature includes reports on: (i) the experimental assessment of the load-carrying capacity of strengthened concrete members while heated or after heating and (ii) material characterization tests on either hot or heated/cooled standalone specimens and joints (composite + substrate).

The following works are referenced herein in line of (i): In Ref. [2], strengthened RC beams were exposed to elevated temperatures while under a sustained load. It was concluded that the strengthening systems could withstand exposure to temperatures of at least 464 °C under the conditions of cold (or possibly mechanical) anchorage regions. Ref. [3] compared the bending response of RC beams that were reinforced with TRM or FRP systems while heated at 150 °C, concluding that the average effectiveness of the TRM system decreased by 45% in comparison to the effectiveness at ambient temperature, while the FRP one totally lost its effectiveness when it was subjected to the same temperature. Ref. [4] examined the effectiveness of TRM and FRP jackets on the shear strengthening of RC beams while they were heated at various temperatures. According to their findings, the effectiveness of the FRP jackets dropped dramatically when increasing the temperature from 100 °C to 150 °C, while the effectiveness of the TRM jackets was marginally affected due to their exposure to 100 °C, 150 °C, and 250 °C. In Ref. [5], a strengthened RC slab strip was subjected to flexure under a constant service load while it was exposed to fire for two hours according to a European standard curve, [6]. It was found that the slab could carry the applied load for the full two hours during which the composite mesh reached a temperature of about 440 °C. Ref. [7] applied concentric axial compression to jacketed concrete cylinders with TRM or FRP systems while heating to steady-state temperatures between 20 °C and 400 °C. The main outcome was that the effectiveness of FRP decreased considerably, but did not vanish, with increasing temperatures, in particular within the region of the glass transition temperature of the epoxy resin/adhesive. Conversely, the TRM system demonstrated a superior performance over the FRP one at 400 °C. Ref. [8] studied the residual compressive strength of confined cylinders with a TRM jacket after their exposure to a cycling thermal exposure up to various temperatures i.e., 100 °C and 200 °C. They showed that the axial strength and the respective confinement ratio of the cylinders slightly reduced after exposure to 100 °C, while the same parameters decreased by 67% and 20%, respectively, after conditioning at 200 °C. Regarding masonry members, the literature is limited to the study of Ref. [9] where the post-fire resistance of masonry walls strengthened with a combined system of TRM and thermal insulation against out-of-plain bending is experimentally tested. When the insulation material was sandwiched between the wall and the TRM system, the composite reached 870 °C at an exposure temperature 1006 °C and was severely damaged. Fired specimens that were furnished with this system exhibited zero or minimal flexural strengths in comparison to the counterpart unfired ones. On the contrary, when the TRM system was sandwiched between the wall and the heat shielding, the walls retained most of their unfired flexural strength. Based on the aforementioned studies, it is concluded that the TRM reinforcement enhances the resistance of strengthened structural members against elevated/high temperatures or fire and preserves its integrity. In addition, most of these studies underline the crucial role of TRM overlays’ adequate anchorage on the substrate for the protective action of the composite material to be fully developed.

As far as works in line of (ii) are concerned, those employing standalone specimens for material characterization purposes are of rather limited use for designing strengthening interventions with TRM and they evade the scope of the current study. They can be used, though, for comparative purposes between different strengthening systems. On the other hand, limited studies have investigated the residual shear bond strength of TRM-to-masonry/brick [10,11,12,13] or TRM-to-concrete joints [14] after exposure to elevated/high temperatures or during the heating of TRM-to-concrete joints [15]. These studies have been thoroughly reviewed by Ref. [16]. Based on Ref. [16], it is concluded that: (i) there is a lack of commonly accepted testing protocols for the investigation of the mechanical performance of TRM systems (for use as strengthening systems) under or after exposure to elevated/high temperatures, (ii) the wide variety of TRM systems renders any comparison effort a challenging task, and (iii) the inherent variability of shear bond test results, in particular, calls for testing a large number of identical specimens for each test.

The optimum exploitation of externally bonded TRM products (typically attained when the textile reaches its tensile strength) is achieved under the condition that premature debonding of the overlay(s) from the substrate or interlaminar debonding of the textile from the matrix are absent when strengthened structural elements are loaded. Therefore, the study of the TRMs-to-substrate bond response is of crucial importance and many experimental studies have already been dedicated to the topic concerning either masonry (e.g., [17,18,19,20]) or concrete substrates (e.g., [21,22,23,24]).

To render TRM products more eco-friendly (by eliminating ordinary Portland cement usage), some researchers have already developed alkali-activated mortars that can be used as matrices. In addition, there are already examples of TRM systems that are based on alkali-activated mortars that have exhibited good resistance against exposure to high temperatures. For example, in Ref. [25] the researchers combined bidirectional basalt, glass, or carbon uncoated textiles and unidirectional steel cords of various geometries (i.e., of different yarns’ or cords’ distance) with a mortar that was based on both metakaolin and furnace slag creating six textile-reinforced alkali-activated mortar (TRAAM) systems. The compressive strength of the AAM increased logarithmically with time reaching 45 MPa after one year and remained almost unaffected after exposure to 50 freeze-thaw cycles and to temperatures up to 1000 °C. In addition, Ref. [26] studied the flexural response of AAM panels –comprising of an alkali-activated mortar that was based on blast furnace slag that was reinforced with basalt textile and steel fibers post their exposure to elevated temperatures (400 °C, 600 °C, and 800 °C). They reported that the flexural strength of the specimens after exposure to 800 °C for 1 h or 2 h decreased by 90% and 92%, respectively. The decrease of the flexural performance as the temperature and the exposure duration increased took place due to the decomposition of the matrix and the deterioration of the textile–matrix bond. It is also interesting to mention that a TRM system that was based on alkali-activated mortar was developed by Ref. [27] for energy retrofitting of masonry panels. The alkali-activated mortar which contained fly ash and metakaolin as precursors and expanded glass as aggregates was combined with a GFRP mesh and was compared with a “conventional” TRM system comprising of a lime-based matrix. The alkali-activated textile-reinforced mortar presented lower thermal conductivity than the conventional one without compromising its mechanical characteristics.

The current study is focused on the experimental investigation of the effect of elevated temperatures on the residual shear bond strength of four TRM systems when they are applied as external reinforcement on masonry elements. To this purpose, wall prisms were unilaterally reinforced with two strips of a dry AR glass fiber textile which were embedded in various matrices in terms of chemical composition and density, i.e.,: two cementitious matrices, namely a normal-weight (Textile-Reinforced Cement-based Normal-weight Mortar-TRCNM) and a lightweight (Textile-Reinforced Cement-based Light-weight Mortar-TRCLM) one; and two counterpart alkali-activated matrices (Textile-Reinforced Alkali-Activated, Normal-weight Mortar-TRAANM) and (Textile-Reinforced Alkali-Activated Light-weight Mortar-TRAALM). The reinforced specimens were exposed to 200 °C and to 400 °C for 1 h and, after their heating treatment, they were tested using the single-lap/single-prism (SL/SP) setup. The bond response of the heated specimens was compared with the response of the reference ones (unheated specimens sharing the same TRM system). The mortars that were used as matrices for the development of the aforementioned TRM systems shared comparable flexural strengths per density class. In addition, per binder type, the mortar composition in terms of constitutive materials’ volumetric ratios (excluding water) was identical between the mortars of different density classes. It is highlighted that this is one of the first studies that investigates alkali-activated and/or lightweight matrices for the design of TRM strengthening systems that can also offer protection of existing masonry members exposed to elevated temperatures.

## 2. Materials and Methods

### 2.1. Substrate

Wall prisms with horizontal joints were used for the simulation of the masonry substrate. Each prism comprised of 5 ridge-faced perforated fired clay bricks with nominal dimensions 190 mm × 83 mm × 58 mm (as in length × width × height) and 4 mortar joints with a thickness of 10 mm. The compressive strength of the bricks that was parallel and perpendicular to the perforations was equal to 8.9 MPa and 3.7 MPa, respectively, while the joints consisted of an M10 general purpose masonry mortar according to the EC 6 classification [28]. The mixture of the joints’ mortar contained cement (CEM II 32.5 N), lime, and sand in proportions 1:0.5:5, by volume. The compressive strength and the elastic modulus of the wall prisms perpendicular to their joints was defined according to the recommendation LUMB1 of RILEM TC 76 [29] and were found equal to 5.8 MPa (CoV 10%) and 3.2 GPa (CoV 19%), respectively.

### 2.2. TRM Overlays

A total of four composite materials were used as strengthening overlays consisting of the same textile and different matrices. The textile that was used was a balanced woven grid of dry AR-glass fiber yarns with a weight equal to 300 gr/m^2^ and a mid-yarn spacing that was equal to 17 mm. The mechanical characteristics of the textile were determined through tensile tests of 5 strips with 7 longitudinal (load-aligned) yarns each, partially following the provisions of EN ISO 13934-1 [30]. According to the experimental results, the tensile strength (*f_tex_*) and the elastic modulus (*E_tex_*) of the textile were found equal to 505 MPa (CoV 11%) and 83 GPa (CoV 17%), respectively.

A total of two cement-based and two alkali-activated mortars were used as matrices. For both types of mortars (cement and alkali-activated), two variations were formulated through the replacement of natural normal-weight aggregates (different for cement and alkali-activated mortars) with artificial lightweight ones (namely, expanded glass) of identical granularity. Hence, four mortars were designed and produced to serve as matrices. Table 1 provides information on the composition, the air-dry density, and the flexural/compressive strength (both determined at 28 days according to EN 1015-11 [31]), for all the mortars. Per binder type, the mortar composition in terms of constitutive materials’ volumetric ratios (excluding water) was identical between the mortars of different density classes. Additionally, per the density class, the flexural strengths were proved to be comparable. Finally, PVA fibers (known for their good durability in alkaline environments) were used in lightweight mixes to counterbalance the toughness that was lost due to the use of expanded glass aggregates and in normal-weight ones for shrinkage cracking control.

**Figure 1:**
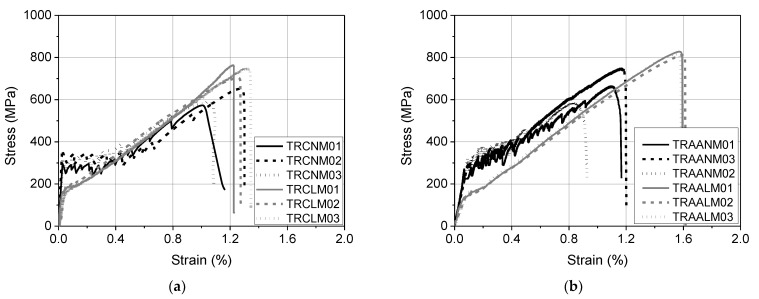
The axial tensile stress versus the axial tensile strain curves of: (**a**) textile-reinforced cementitious mortar and (**b**) textile-reinforced alkali-activated mortar coupons (stress is calculated by dividing with the load-aligned fibers’ cross section).

**Figure 2:**
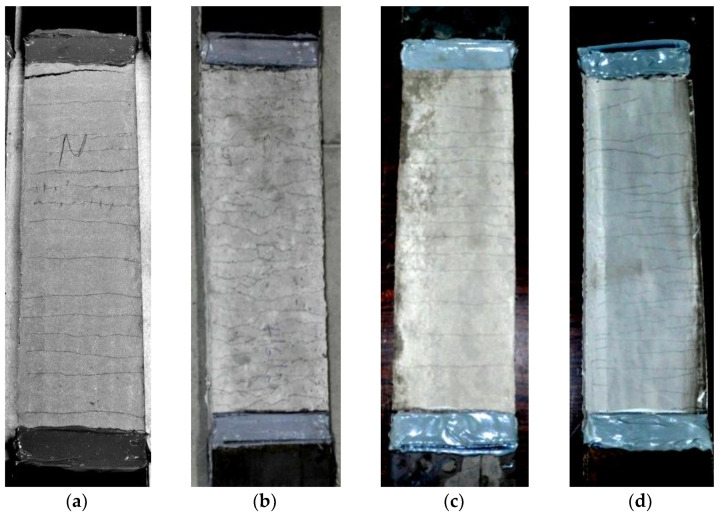
The coupons after tensile testing: (**a**) TRCNM, (**b**) TRCLM, (**c**) TRAANM, and (**d**) TRAALM.

The constitutive law in tension of the TRM systems was determined partially following the procedure that was described in AC434 ICC-ES [32]. The axial stress versus the axial strain curves for each system are presented in Figure 1 based on the results of three identical specimens (coupons with prismatic section), per system. It is noted that each coupon comprised of two textile strips that were sandwiched between three mortar layers to represent the reinforcement that was adopted for the SL/SP specimens (see Section 2.4). The notation of the TRM coupons has the form TRxyMi, where x stands for the type of binder of each mortar, namely C for cement-based and AA for alkali-activated; y denotes its density, namely N for normal-weight and L for lightweight; and i is the number of each specimen that belongs to the same group. The first crack stress (*f_FCR_*) along with the tensile strength (*f_TRM_*) and the corresponding axial strains (*ε_FCR_*, *ε_TRM_*) are listed in Table 2, while photos of the representative specimens from each group after test termination are shown in Figure 2. The TRCNM and TRAANM coupons present a strain-hardening response and their stress-strain curves could be idealized as trilinear and bilinear up to failure, respectively. Their crack pattern was dense with the cracks’ positions almost coinciding with the transversal yarns of the textile strips. All of the lightweight coupons, namely the TRCLM and TRAALM ones, were also characterized by a strain-hardening behavior and their stress-strain curves could be idealized as bilinear up to failure. The crack pattern of the lightweight coupons was denser and more random than the one of the normal-weight coupons owed to the cracks’ bifurcation. This fact is attributed to the higher fiber volume fractions that were incorporated in the lightweight matrices than in the respective normal-weight ones; not only textile fibers but also the chopped fibers added in the former matrices participate in the stress-bridging mechanism. In all cases, failure was due to the slippage of the load-aligned fibers from the mortar with simultaneous fibers’ fracture during the enlargement of a previously created mortar crack. The exploitation degree of the textile (ratio of TRM tensile strength over textile tensile strength) was invariably larger than unity due to the presence of discrete fibers in the matrices. Per the binder type, the different fiber volume fractions resulted in different degrees of textile strength exploitation. The alkali-activated matrices proved to be good alternatives to cement-based ones for the production of strain-hardening inorganic matrix composites. Data regarding the behavior of the TRM systems that were developed in this work under tension is provided mainly for the sake of completeness. The tensile test results cannot provide a solid basis for the interpretation of the response of TRM/substrate joints under shear bond conditions.

### 2.3. Experimental Program

The experimental program is presented in Table 3. In total, 36 shear bond specimens were constructed. Half of them, i.e., 18 specimens, were furnished with cement-based TRM systems and the rest with alkali-activated ones. Among the specimens with TRM overlays sharing the same binder, 9 specimens comprised of a normal-weight matrix and the rest a lightweight one. In each test group (wallettes receiving the same type of TRM overlay), 3 specimens served as reference (unheated) ones whereas the rest were tested after heating at 200 °C or 400 °C for 1 h (3 for each exposure temperature). The notation of the specimens has the form TRxyMz_i, where x stands for the type of binder in the mortar, namely C for cement and AA for alkali activated; y stands for its density, namely N for normal-weight and L for lightweight; z stands for the exposure temperature; while i represents the number of each specimen in a group of identical ones.

### 2.4. Specimens’ Construction

**Figure 3:**
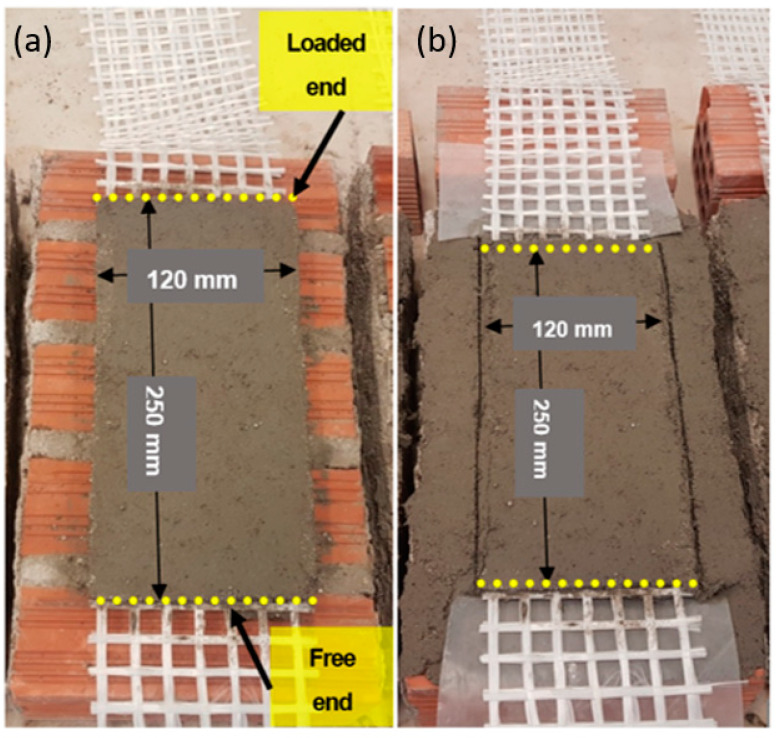
Specimens after casting of the TRM overlay: (**a**) reference and (**b**) intended for exposure to elevated temperature.

The shear bond specimens consisted of stack-bonded wall prisms that were unilaterally reinforced with TRM overlays (Figure 3). Specifically, each wall was furnished with two textile strips that were sandwiched between three matrix layers of equal thickness, namely 4 mm. The TRM overlay had a bond length that was equal to 250 mm so that the textile strips could be considered as adequately anchored on the wall. The width of the TRM overlay, which was centrally placed across the wall’s width, was equal to 120 mm given that each textile strip consisted of 7 longitudinal (load-aligned) yarns. It is also noted that the location of the strip along the wall’s height was chosen in a way that ensured that the maximum number of joints were in contact with the overlay. The textile strips that projected from the overlay’s loaded end had a length that was equal to 400 mm; part of them also projected from the free end (Figure 3). Especially in the case of the specimens that were intended for exposure to elevated temperatures, the wall surface (TRM side) was covered by the same mortar used as matrix in the composite to simulate real-life conditions during heating (Figure 3b). The curing process of the specimens after TRM casting included covering of the overlays cover with wet burlaps for 7 days and subsequent storage of the specimens in a chamber at 22 °C ± 2 °C and Relative Humidity 63% for 21 days.

### 2.5. Thermal Treatment

#### 2.5.1. Drying

All shear bond specimens were dried at 40 °C for 24 h. To this purpose, they were inserted in an electric forced air furnace. The aim of specimens’ drying was to bring them to a state of approximately equal moisture content. After their removal from the furnace, they were wrapped with a PVC membrane to prevent moisture exchange with the atmosphere. 

#### 2.5.2. Heating

**Figure 4:**
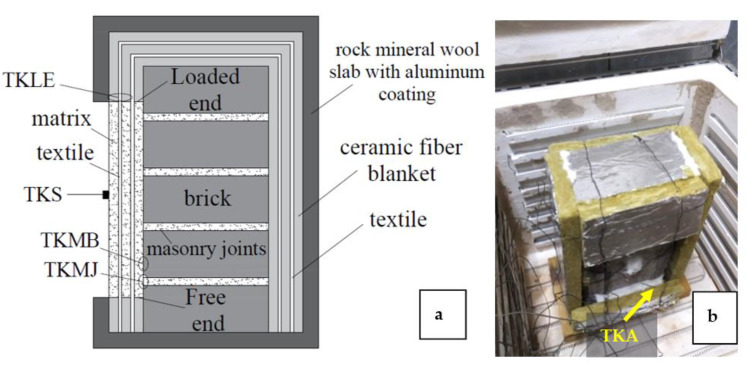
The insulated specimens: (**a**) drawing of its section view and (**b**) photos after being placed in the furnace.

The exposure of the specimens to elevated temperatures was achieved through their insertion in an electric furnace that was equipped with resistance wires on the entire surface of its perimeter walls and on its bottom. For the projecting parts of the textile strips to be protected during heating, a combination of insulation materials was adopted, i.e., a ceramic fiber blanket and rock mineral wool slabs with aluminum coating. A section view of the insulated specimens is presented in Figure 4 along with a photo of a specimen inside the furnace before the commencement of the heating scheme. The specimens were placed upright inside the furnace while the distance of the TRM’s surface from the resistance wires was kept constant and equal to 250 mm. A total of four K-type thermocouples were used as heat sensors being insulated against radiation heating. Their points of application are presented in Figure 4a; these were: the loaded end (TKLE), the TRM–brick and/or the TRM–joint interface (TKMB and/or TKMJ), and the surface of the TRM overlay (TKS). An additional thermocouple had been adjusted close to the specimen for the monitoring of the air temperature of the furnace (TKA, Figure 4b). The heating rate of the furnace was kept constant for both the maximum target exposure temperatures (200 °C and 400 °C) and was equal to 7 °C/min. After the attainment of the target temperature, the air temperature of the furnace was kept constant for 1 h. Each specimen was left to cool down freely inside the furnace; to this purpose its door remained closed until the chamber air temperature reached the ambient temperature. It is noted that due to the absence of a commonly accepted testing protocol, the main parameters of the heating process were selected based on the relevant studies that were mentioned earlier (i.e., [10,11,12,14]). The target exposure temperature was selected to be 100 °C lower and higher than 300 °C (which was the maximum temperature that was investigated in [10,11,12,14]) while the exposure duration that was selected accounted for the minimum one that was referenced in [10,11,12,14].

**Figure 5:**
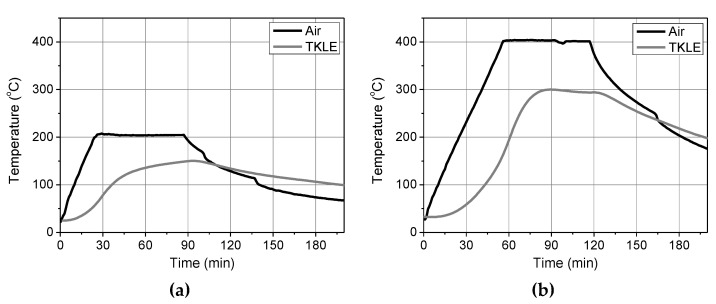
The temperature profiles as recorded by thermocouples during heating at (**a**) 200 °C and (**b**) 400 °C.

The effectiveness of the thermal insulation in preserving the integrity of the projecting pieces of the textile at the loaded end was assessed through the following procedure. A total of six “dummy” wall prisms that were furnished with TRM strips were insulated for exposure to 200 °C and 400 °C (3 specimens per exposure temperature) and underwent the same heating protocols as those that were applicable for the rest of the test specimens. After completion of heating, the projecting parts of the textile strips (2 per specimen) were cut and tested in tension according to the same testing procedure that was followed for the unheated textile strips (see Section 2.2). The average values of the tensile strength (*f_tex_*) and the elastic modulus (*E_tex_*) corresponding to the heated textile strips that were located in between the bottom and the intermediate mortar layer (named as “inner”) and in between the top mortar layer and the intermediate one (named as “outer”) are listed in Table 4 for all the exposure temperatures, including ambient. The temperature that was developed on the outer strip during the specimen’s heating was recorded through a K-type thermocouple that was placed at the same point as the TKLE one (Figure 4a). The record of the aforementioned thermocouple along with the air temperature of the furnace, recorded by TKA, are presented in Figure 5 for both the exposure temperatures. In the case of heating at 200 °C, the maximum temperature that was developed on the outer textile strip was around 150 °C (see Figure 5a) while in the case of heating at 400 °C it was around 300 °C (see Figure 5b). According to the results of Table 4, the specimens’ exposure to 200 °C slightly enhanced the mechanical characteristics of the projecting (but insulated) textile strips. On the other hand, exposure to 400 °C had a clear negative effect on the tensile strength of the textile, especially that of the outer strip. The specimens’ exposure to 200 °C resulted in the softening of the sizing of individual filaments comprising the part of the yarn sleeve that was close to the heating front. This led to filaments’ “fusing” and to better exploitation of the tensile strength of each yarn. In the case of specimens that were exposed to 400 °C, the sizing was completely lost (burned), causing damage to the filaments and compromising the textile’s tensile strength.

**Figure 6:**
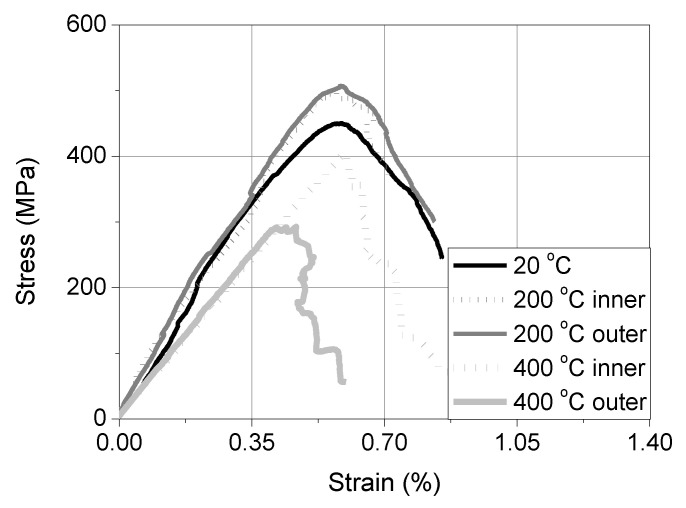
The representative curves of axial stress versus the strain of textile strips under tension.

The representative tensile stress-strain curves of all the textile strips [unheated and heated (but heat-protected)/inner and outer] are presented in Figure 6. The investigation that is described above aimed at exploring the possible limitations of shear bond test configurations, employing heated specimens, in which the shear load is transferred to the substrate/composite interface through pulling the textile away from the matrix (as in the case of single-lap/single-prism setup). Should the heated projecting textile (no matter the heat protection that is applied) pose the weak link during shear bond loading then this type of test setup must either be redesigned or abandoned.

### 2.6. Shear Bond Tests

**Figure 7:**
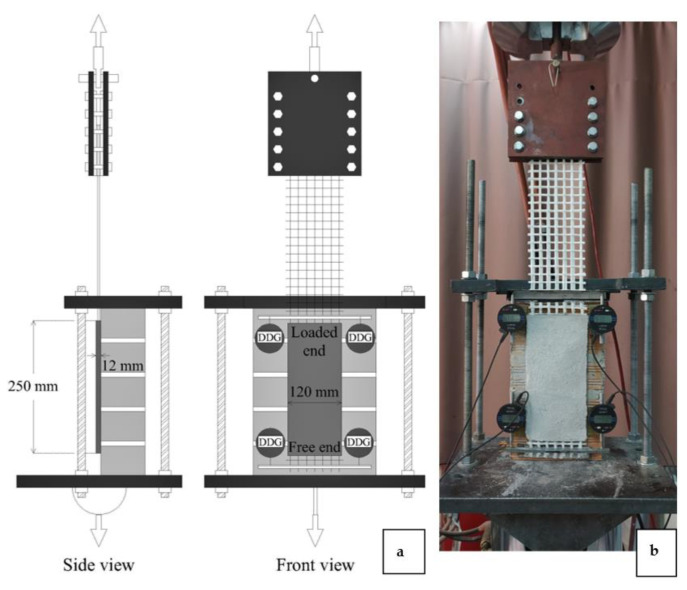
(**a**) A drawing and (**b**) a photo of the SL/SP setup.

For the implementation of the shear bond tests, the single-lap/single-prism (SL/SP) setup was adopted (Figure 7) that was based partially on the recommendation of RILEM TC 250-CSM [33]. The choice of the setup was dictated by the space limitations of the furnace that was used for the specimens’ heating. Each reinforced wall was fitted in a steel frame which, in turn, was connected to the moving part of a servo hydraulic testing machine (Figure 7). The textile strips that were projecting from the overlay’s loaded end were connected to the fixed part of the machine through a joint that provided full in-plane and partial out-of-plane rotation capacity. To this purpose, the extremities of the projected strips had been sandwiched between three glued-on fiber-reinforced polymer (FRP) tabs two days prior to testing. The connection was performed by clamping the FRP tab between two bolted hinged steel plates. During the testing, the projecting strips were being pulled away from the TRM overlay following a piston displacement-controlled mode with a rate that was equal to 0.005 mm/s. The instrumentation of the specimens comprised 4 digital dial gauges (DDG) that were glued on the wall. Of them, two had been placed close to overlay’s loaded end (DDGLE) and the rest were close to the free end (DDGFE). Each pair of gauges was acting against an aluminum plate that was glued on the second transversal yarn of the strips that were projecting from each overlay’s end (Figure 7). The records of DDGLE represented the textile’s relative displacement in relation to the substrate while the records of DDGFE represent the textile’s slip from the matrix. It is noted that only one specimen from each group of identical ones was equipped with DDGFE.

## 3. Experimental Results and Discussion

### 3.1. Visual Effect of Heating

**Figure 8:**
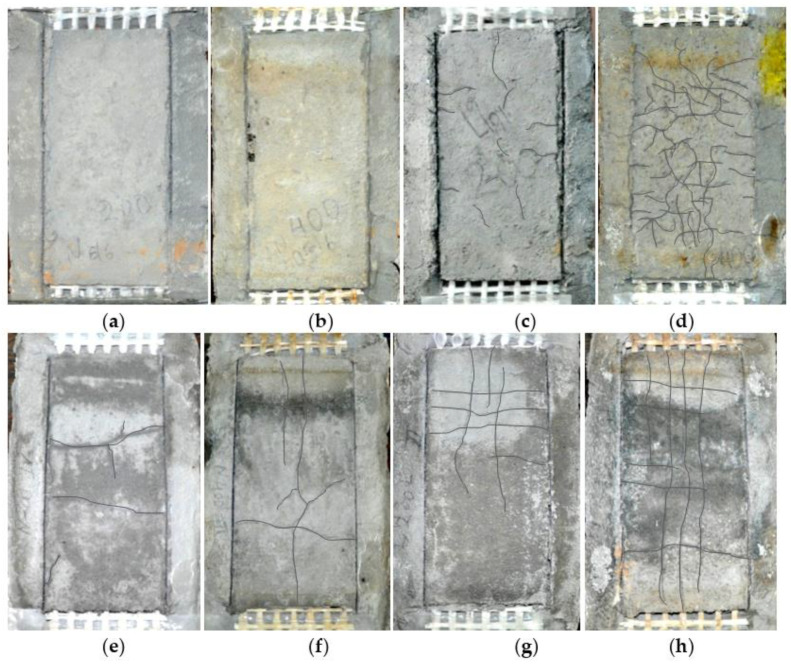
SL/SP specimens after their heating: (**a**) TRCNM200, (**b**) TRCNM400, (**c**) TRCLM200, (**d**) TRCLM400, (**e**) TRAANM200, (**f**) TRAANM400, (**g**) TRAALM200, and (**h**) TRAALM400.

The majority of the specimen’s fine cracks were formed on the top mortar layer of their TRM overlay after the completion of their thermal treatment. A visual inspection of the cracks revealed that their depth was less than or equal to the thickness of the top mortar layer of the overlay. It is noted that no shrinkage cracks had been noticed either before or after the drying process of the specimens. The PVA fibers in all the TRM strips that were bonded on the heated specimens seemed to remain unaffected after exposure to 200 °C; the color of those fibers that were visible on the surface of the top mortar layer turned to grey after exposure to 400 °C. The detailed presentation of the cases where cracks that formed due to heating is carried out in Table 5. Additionally, photos of the specimens after their heating are listed in Figure 8. Based on these, it is observed that in the case of alkali-activated matrices the cracks almost run along the yarns while in the case of cementitious matrices, the cracks (where present) are randomly distributed. Map cracking in TRCLM specimens is an indication of high evaporation rates during heating. The cracks that were coincidental with the fiber yarns in TRAANM and TRAALM specimens are signs of both a higher degree of mortar penetration in the dry fiber yarns (than the one that was achieved in cement-based TRMs) and thermal shrinkage during heating.

The temperature values that were recorded by the thermocouples TKLE, TKS, TKMB, and TKMJ (Figure 4a) at the beginning and at the end of the heating duration when furnace temperature was kept constant and equal to each examined value (i.e., 200 °C and 400 °C for 1 h) are listed on Table 3 and discussed in Section 4.

### 3.2. Failure Mode

All the specimens failed due to fibers’ rupture. A critical amount of sleeve (well-bonded to mortar) fibers failed leading to the loss of load-carrying capacity as the inner (core/unbonded) fibers in the yarns, which were left intact, slipped through the mortar failing to result in strength recovery. Regarding all the examined TRM systems, as the exposure temperature increased, more sleeve fibers failed leading to the attainment of lower maximum bond load. Finally, it is noted that the cracks that were formed due to specimens’ heating on the top mortar layer of the TRM overlay did not increase in number during shear bond testing.

### 3.3. Stress vs. Relative Displacement Curves

The experimental results of the shear bond tests are listed in Table 3 in terms of: (i) the maximum textile axial stress (*σ_max_*) that was computed as the ratio of the maximum load that was carried by the TRM overlay to the cross sectional area of the longitudinal fibers which is equal to 13.09 mm^2^, (ii) the corresponding relative displacement of the textile with respect to the wall prism (*d_r,max_*) being equal to the average of the readings from the DDGLE, (iii) the corresponding textile slip from within the mortar (*s_max_*) being equal to the average of the readings from the DDGFE, and (iv) the textile’s exploitation ratio that was calculated by dividing the *σ_max_* by *f_tex_* values that corresponded to each examined temperature (see Table 3 and Table 4). It is noted that an even load distribution was assumed between the two textile strips of the TRM overlay as well as among the longitudinal yarns of the same strip.

**Figure 9:**
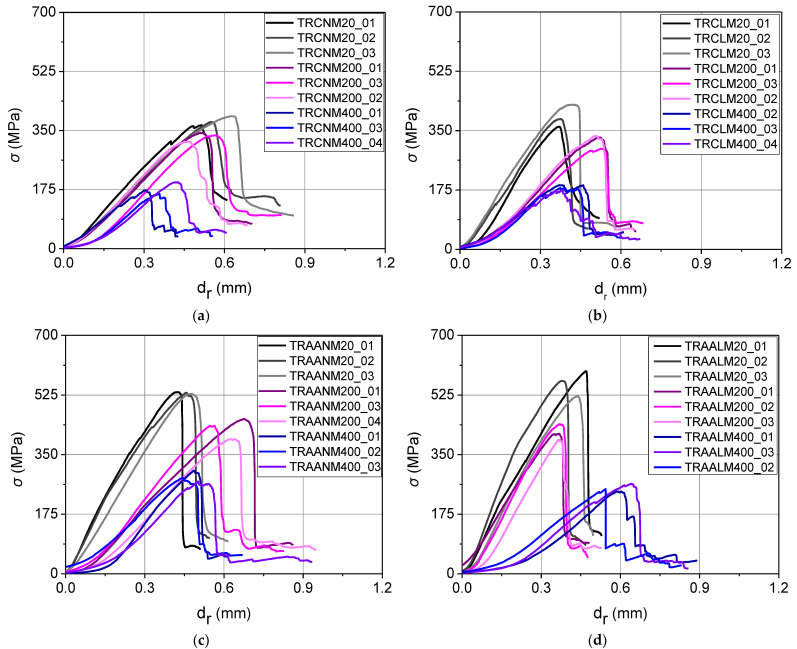
The response curves –textile axial stress (*σ*) versus the textile relative displacement with respect to the substrate (*d_r_*) – of the SL/SP specimens that were reinforced with TRM overlays comprising cementitious (**a**) normal-weight or (**b**) lightweight matrix and alkali-activated (**c**) normal-weight or (**d**) lightweight matrix.

The specimens’ response during the shear bond tests is visualized through the curves of the textile axial stress (*σ*) versus the relative displacement of the textile with respect to the wall prism (*d_r_*) which are presented in Figure 9 for all the examined TRM systems.

The response curves (Figure 9) present a quasi-linear ascending branch which deviated from linearity close to the maximum. The inclination of the ascending branch of the response curves represents the “stiffness” of the textile-to-matrix interface. A decreasing inclination of this branch with increasing temperature reflects the effect of heating on the matrix and hence on its bond with the fibers. The latter is directly related to the extension and morphology of the thermal cracking that was induced in the matrix. Indeed, in TRCNM specimens (which remained uncracked after heating), the inclination of the ascending branch of the textile axial stress vs. relative displacement curves was kept unchanged for increasing exposure temperature. For all the other specimens (which developed some type of cracking) the inclination generally decreased for increasing temperature. An indication of thermal damage in the projecting textile (although thermally protected) is the “drag” in the curves, that is, the delay in the *σ* increase with increasing relative displacement at the onset of the test.

The post-peak response of the curves is characterized by an abrupt load drop due to fibers’ rupture, leading to specimens’ failure (see Section 3.2). After the attainment of the maximum load, the load distribution among the longitudinal yarns does not remain uniform. The residual load, if present, varies depending on the number of load-aligned fibers that remained intact. The nature of this phenomenon is highly stochastic, therefore, comments based on the residual load values cannot lead to safe conclusions.

### 3.4. Other Performance Markers

**Figure 10:**
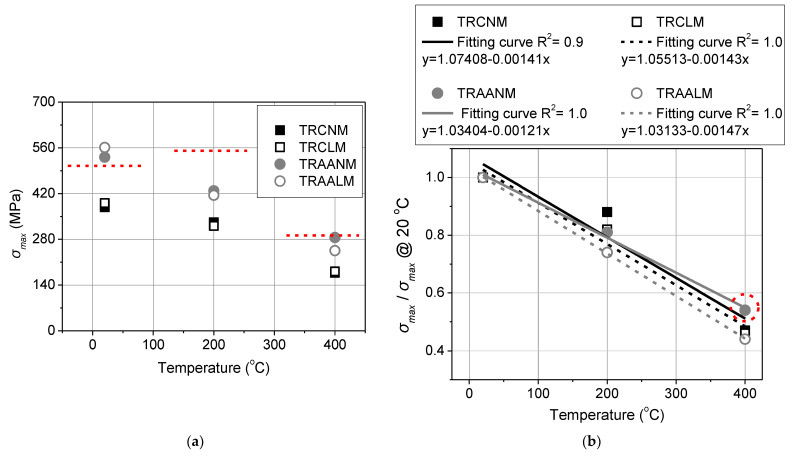
(**a**) The maximum textile axial stress (*σ_max_*) and (**b**) the ratio of the maximum textile axial stress of the heated to the reference SL/SP specimens versus elevated temperatures.

The impact of the specimens’ heating on the textile–matrix bond strength (note that based on the specimens’ failure mode, the interface of interest is that between the textile and the matrix) is depicted in Figure 10a where *σ_max_* values of both the reference and the heated specimens are presented versus the examined exposure temperatures. Based on the results that are listed in Table 4, the (outer) textile strength (*f_tex_*) corresponding to each exposure temperature is also plotted in Figure 10a with a dotted red line. For the ambient conditions, *f_tex_* does not differ between inner and outer textile layers. In the heated specimens, *σ_max_* values that were found below this line indicate that failure was not attributed to thermal damage of the projecting (mortar-free) textile. In the unheated specimens, *σ_max_* values found: (a) below this line denote textile exploitation ratios that are less than unity (which is the norm in cement-based TRMs) and (b) above this line denote textile exploitation ratios that are larger than unity (which is attributed to the high penetrability of alkali-activated matrices into fiber yarns and increased fiber bonding capacity-see TRAANM and TRAALM). Figure 10b presents the normalized residual bond strength (the ratio of heated over unheated bond strength) as a function of the temperature for each TRM system. 

The residual bond strength of TRM/masonry and TRAAM/masonry joints decreases as exposure temperature increases (Figure 10a). Specimens that were furnished with TRMs sharing the same binder exhibited (per exposure temperature) almost identical *σ_max_* values irrespective of their density class. Although not directly comparable (different binder granulometry and paste structure but comparable flexural strengths per density class), the alkali-activated textile reinforced mortars outperformed their cement-based counterparts in terms of *σ_max_*, at every temperature. When moving from cement-based systems to alkali-activated ones the increase in shear bond strength was found to be approximately equal to 40%, for specimens that were tested in ambient conditions without prior heating. After heating at 200 °C and 400 °C, the increase in shear bond strength of the cooled-down specimens was equal to 30% and at least 35%, respectively (35% and 60%, for lightweight and normal-weight matrices).

For all the heated specimens, the *σ_max_* values were lower than the tensile strength of the projecting textile that was heated at the same temperature (outer layer, taken as the worst-case scenario); this denotes that failure was the result of matrix thermal damage. It should be noted, however, that *σ_max_* values of TRAANM400 specimens were marginally lower than *f_tex_* after heating at 400 °C. Therefore, the residual bond strength of this type of TRM, as measured herein, should be taken as a lower bound. It is highly probable that the thermal resistance of the alkali-activated normal-weight matrix (and, consequently, the residual post-heating shear bond strength thereof) is higher than that which is shown here.

In terms of residual bond strength that is expressed as a ratio of the bond strength after heating over the reference (the unheated) one (Figure 10b), all the systems retained close to 50% of their original strength (namely, 47%, 54%, and 44% for TRCNM and TRCLM, TRAANM and TRAALM, respectively) after heating at 400 °C. The ratio decreased linearly with increasing temperature for all the TRM systems.

**Figure 11:**
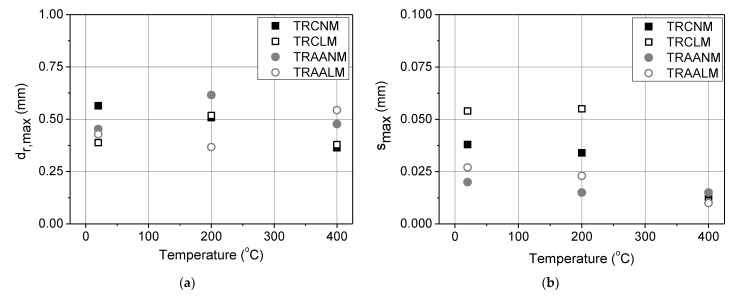
(**a**) The relative displacement (*d_r_,_max_*) and (**b**) slip (*s_max_*) corresponding to the maximum textile axial stress versus the exposure temperature.

The values of relative displacement and slip that correspond to the maximum attained bond load, i.e., *d_r,max_* and *s_max_*, respectively, versus the examined exposure temperature are presented in Figure 11. It is observed that for all the matrices, *d_r,max_* does not follow a consistent trend with increasing temperature. Nevertheless, per matrix type, the maximum differences of *d_r,max_* values between the different exposure temperatures are restricted to less than 50%. The *s_max_* values of the reference specimens are comparable to those of the specimens that were exposed to 200 °C. For all the matrix types, the *s_max_* values of the specimens that were exposed to 400 °C were almost identical and of a very low magnitude; this is probably attributable to the effect of the heating on the projecting textile at the loaded end.

Last, a short discussion on the temperatures that were recorded by the thermocouples is provided. It is noted that at the end of the 1-h heating period, the specimens that were subjected to 200 °C had undergone a total heating time of 1.5 h (the heating ramp included) whereas the respective period for specimens subjected to 400 °C was equal to 2 h (see Figure 5). Another useful remark is that although unidirectional heating conditions were meant to be in effect, the thermal insulation was not able to keep the masonry prisms from increasing their temperature. From sparse measurements using a thermocouple that was placed at the rear surface of the wallettes (masonry/insulation interface) it was shown that the temperature reached 250 °C at the end of the 1 h heating period at 400 °C. This means that both the masonry components (bricks and masonry joints) were heated from all sides from which only one was partially covered by the TRM overlay.

**Figure 12:**
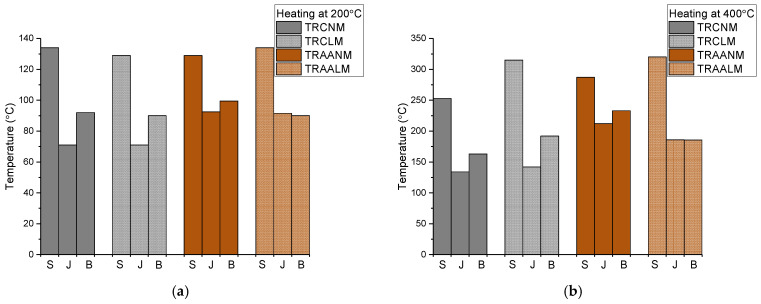
Temperatures at the TRMs’ surface (S), the TRM/masonry joint interface (J), and the TRM/brick interface (B) in specimens that were heated at: (**a**) 200 °C and (**b**) 400 °C. (In case more than one temperature value exists for identical specimens, the average values are considered).

Figure 12 shows that, for all the examined TRM systems, the values of TKS (surface thermocouple), TKMB (thermocouple on the TRM/brick interface), and TKMJ (thermocouple on the TRM/masonry joint interface) that were recorded after 1 h of heating at 200 °C and 400 °C. The symbols “S”, “J”, and “B” in Figure 12 stand for “surface”, “joint”, and “brick”, respectively.

- The surface (TRM) temperature for all the specimens that were heated at 200 °C is the same (approx. equal to 130 °C). Heat protection of the masonry at the interface of the TRMs with the brick is also comparable between the systems. The same does not apply for the TRM/masonry joint interface, however. In this case, the cement-based systems provided better heat protection than the alkali-activated ones (this was also observed when heating at 400 °C–see further below for a plausible explanation). Different temperature readings (per TRM type) at the TRM/brick and TRM/masonry joint interfaces (present in all the specimen groups but the TRAALM ones) are owed to the stereoscopic heating of the masonry components and the different thermal properties of the bricks and masonry mortar.
- The surface (TRM) temperature for specimens that were heated at 400 °C seemed to depend upon the density class. TRMs with lightweight matrices developed: (i) identical surface temperatures regardless of the binder type that as used, and (ii) higher surface temperatures with regard to the TRMs comprising of normal-weight matrices of the same binder type. The latter is probably owed to the evaporation of the remaining moisture and chemically-bound water with vapors increasing the surface temperature. Nevertheless, per type of binder, the lightweight matrices resulted in either comparable (cement-based systems) or better (alkali-activated systems) heat protection at the TRM/masonry interface. As in the case of heating at 200 °C, the different temperatures were recorded (per TRM type) at the TRM/brick and TRM/joint interfaces for all the specimen groups but the TRAALM ones. The distinct behavior of alkali-activated lightweight matrices in this respect deserves further investigation to exclude the possibility of coincidental results. The fact that temperatures that were given by TKMJ for TRAALM and TRAANM were generally larger than the ones for TRCNM and TRCLM can possibly be explained as follows. In alkali-activated pastes, the weight loss occurs at temperatures below 300 °C; this phenomenon is related to the evaporation of chemically-bound water and to the significant thermal shrinkage. The latter occurs voluminously, hence, also thickness-wise resulting in decreased thermal insulation of the masonry block. Thermal cracking (more severe for alkali-activated mortars) also creates thermal bridges.

## 4. Conclusions

In the present study, the effect of elevated temperatures on the residual shear bond strength of four TRM systems that were applied as external reinforcement on masonry elements is experimentally investigated. The TRM systems shared the same dry AR glass fiber textile (two layers) that was combined with four different types of matrices: based on the binder that was used, the matrices are distinguished between cement-based and alkali-activated ones; based on density, the matrices (of the same binder type) are either normal-weight (with normal-weight natural aggregates) or lightweight (with expanded glass aggregates). The matrices shared comparable flexural strengths per density class, while, per binder type, the constitutive materials’ volumetric ratios (excluding water) were identical between the matrices of different density classes. The single-lap/single-prism (SL/SP) specimens were exposed to elevated temperatures (200 °C or 400 °C) for 1 h. After cooling, they were tested under ambient conditions to assess their residual shear bond strength. According to the experimental findings, the following general conclusions are drawn:

- Alkali-activated matrices proved to be good alternatives to cement-based ones for the production of strain-hardening inorganic matrix composites.
- Different types of matrices resulted in different heat-induced cracking potentials. In general, the lightweight matrices were more prone to cracking.
- Exposure to elevated temperatures did not alter the failure mode that was observed during the shear bond testing of unheated specimens which was due to sleeve fibers’ rupture along with core fibers’ slippage from the mortar.
- Despite the fact that the projecting textile was insulated during the specimens’ heating, its tensile strength was compromised after exposure to 400 °C. Nevertheless, for all the heated specimens, the *σ_max_* values were lower than the tensile strength of the projecting textile that was heated at the same temperature; this denotes that failure was the result of matrix thermal damage.
- Should the heated projecting textile pose the weak link during shear bond loading, then the SL/SP test setup must either be redesigned or abandoned.
- The residual bond strength of TRM/masonry joints (TRM being either cement-based or alkali-activated) decreases as the exposure temperature increases.
- Specimens that were furnished with TRMs sharing the same binder exhibited (per exposure temperature) almost identical *σ_max_* values irrespective of their density class.
- The alkali-activated textile-reinforced mortars outperform their cement-based counterparts in terms of *σ_max_*, at every temperature.
- In terms of the residual bond strength that is expressed as a ratio of the bond strength after heating over the reference (unheated) one, all the systems retained close to 50% of their original strength after heating at 400 °C. The ratio decreases linearly with increasing temperature for all TRM systems.
- Per type of binder, the lightweight matrices resulted in either comparable (cement-based systems) or better (alkali-activated systems) heat protection at the TRM/masonry interface.

## Figures and Tables

**Table 1:** The composition, air-dry density, and mechanical characteristics of matrices.

Cementitious Matrix	Normal-Weight	Light Weight
Composition	kg/m^3^	
Portland cement (CEM II 42.5 N)	586	655
Sand (*d_max_* = 2 mm) *	1047 (limestone)	269 (expanded glass)
Silica fume (*d_max_* = 1 μm)	47	52
Limestone filler (*d_max_* = 120 μm)	146	163
Effective water **	344	266
PVA fibers (6 mm)	1.3	13
Superplasticizer	4.1	5
Air-dry density	kg/m^3^	
	2034	1202
Flexural strength at 28 days	MPa	
	7.3	5.7
Compressive strength at 28 days	MPa	
	61.6	23.6
**Alkali-Activated Matrix**	**Normal-Weight**	**Light Weight**
Composition	kg/m^3^	
Metakaolin	325	325
Fly ash	281	281
Ladle furnace slag	63	63
Sand (*d_max_* = 2 mm) *	1113 (siliceous)	250 (expanded glass)
Potassium waterglass	375	375
Potassium hydroxide pellets	56	56
Effective water **	200	225
PVA fibers (6 mm)	3	7
Air-dry density	kg/m^3^	
	1814	1001
Flexural strength at 28 days	MPa	
	6.8	5.3
Compressive strength at 28 days	MPa	
	46.3	16.7

* Water absorption: 3%, 0.2%, and 23% by dry sand mass for the limestone, the siliceous, and the lightweight sand, respectively. ** The water quantity was adjusted during mixing beyond the effective one that was based on the water absorption and the moisture content of each type of sand.

**Table 2:** The mechanical characteristics of the textile reinforced mortar coupons.

Coupon’s TRM System	First Crack Stress	Axial Strain Corresponding to *f_FCR_*	Tensile Strength	Axial Strain Corresponding to *f_TRM_*
	*f_FCR_* (MPa)	*ε_FCR_* (%)	*f_TRM_* (MPa)	*ε_TRM_* (%)
	[CoV]	[CoV]	[CoV]	[CoV]
TRCNM	327 [10%]	0.026 [8%]	604 [7%]	1.105 [13%]
TRCLM	167 [8%]	0.039 [19%]	740 [4%]	1.232 [7%]
TRAANM	297 [8%]	0.084 [18%]	667 [12%]	1.033 [17%]
TRAALM	152 [10%]	0.092 [20%]	819 [1%]	1.556 [3%]

**Table 3:** Experimental results from the SL/SP specimens.

Specimen	TKLE [°C]	TKS [°C]	TKMB [°C]	TKMJ [°C]	*σ_max_* [MPa]	Average [CoV %]	*d_r,max_* [mm]	Average [CoV %]	*s_max_* [mm]	*σ_max_/f_tex_* [%]
TRCNM20_01					366	378 (4)	0.515	0.564 (10)	0.038	72
TRCNM20_02					376		0.549			74
TRCNM20_03					393		0.627			78
TRCNM200_01	54|111	-	-	-	343	332 (4)	0.511	0.508 (11)	0.034	62
TRCNM200_02	54|118	99|134	47|92	34|71	317		0.451			58
TRCNM200_03	67|142	-	-	-	336		0.561			61
TRCNM400_01	157|267	-	-	-	173	178 (10)	0.307	0.364 (17)	0.012	59
TRCNM400_02	177|266	147|253	89|163	77|134	162		0.358			55
TRCNM400_03	106|252	153|256		-	197		0.427			67
TRCLM20_01					362	391 (8)	0.369	0.388 (7)	0.054	72
TRCLM20_02					384		0.375			76
TRCLM20_03					427		0.419			85
TRCLM200_01	59|105	98|129	51|90	36|71	332	321 (7)	0.515	0.518 (3)	0.055	60
TRCLM200_02	74|123	91|129	-	-	334		0.503			61
TRCLM200_03	48|98				297		0.535			54
TRCLM400_01	124|231	-	-	-	190	182 (4)	0.380	0.378 (1)	0.011	65
TRCLM400_02	111|223	220|315	90|192	73|142	181		0.375			62
TRCLM400_03	144|256	217|317		-	174		0.381			60
TRAANM20_01					534	531 (1)	0.423	0.453 (6)	0.020	106
TRAANM20_02					532		0.459			105
TRAANM20_03					529		0.478			105
TRAANM200_01	80|154	85|129	50|101	50|97	454	428 (7)	0.674	0.616 (10)	0.015	82
TRAANM200_02	58|145	-	-	-	396		0.548			72
TRAANM200_03	66|148	89|129	51|98	43|88	435		0.626			79
TRAANM400_01	183|265	-	-	-	303	286 (6)	0.486	0.477 (6)	0.015	104
TRAANM400_02	187|264	-	-	-	282		0.443			97
TRAANM400_03	174|254	184|287	126|233	134|212	271		0.502			93
TRAALM20_01					595	561 (7)	0.469	0.428 (11)	0.027	118
TRAALM20_02					567		0.378			112
TRAALM20_03					522		0.437			103
TRAALM200_01	47|112	90|135	51|87	42|90	411	415 (6)	0.366	0.367 (0.1)	0.023	75
TRAALM200_02	67|146	-	-	-	440		0.367			80
TRAALM200_03	42|116	87|133	44|93	43|93	394		0.367			72
TRAALM400_01	155|236	-	-	-	242	246 (2)	0.586	0.591 (8)	0.010	83
TRAALM400_02	167|299	245|321	89|175	110|177	249		0.544			85
TRAALM400_03	173|283	254|319	99|196	91|195	265		0.642			91

**Table 4:** The mechanical characteristics of the textile after its exposure to the examined temperatures along with the reference values.

Temperature [°C]	Tensile Strength *f_tex_* (MPa) [CoV]	Elastic Module *E_tex_* (GPa) [CoV]
20	505 [11%]	83 [18%]
200 inner strip	551 [14%]	85 [15%]
200 outer strip	569 [16%]	87 [0.1%]
400 inner strip	401 [1%]	74 [7%]
400 outer strip	292 [1%]	71 [12%]

**Table 5:** The cases of crack formation on the top mortar layer of the TRM overlay due to specimens heating.

	Temperature	200 °C	400 °C
System			
**TRCNM**		No cracks	No cracks
**TRCLM**		A limited number of cracks (maximum depth < thickness of the top mortar layer)	Numerous cracks (maximum depth = thickness of the top mortar layer)
**TRAANM**		A limited number of cracks (maximum depth < thickness of the top mortar layer)	Numerous cracks ^1^ (maximum depth = thickness of the top mortar layer)
**TRAALM**		A limited number of cracks ^2^ (maximum depth < thickness of the top mortar layer)	Numerous cracks ^3^ (maximum depth = thickness of the top mortar layer)

^1^ wider than those that were formed at TRCLM after exposure to 400 °C. ^2^ more than those that were formed at TRAANM after exposure to 200 °C. ^3^ more than those that were formed at TRAANM after exposure to 400 °C and wider than those that were formed at TRCLM after exposure to 400 °C.

## References

[1] Bournas D., Triantafillou T. (2016). Strengthening of existing structures: Selected case studies. Textile Fibre Composites in Civil Engineering.

[2] Bisby L., Stratford T., Hart C., Farren S. (2013). Fire performance of well-anchored TRM, FRCM and FRP flexural strengthening systems. Advanced Composites in Construction.

[3] Raoof S.M., Bournas D.A. (2017). TRM versus FRP in flexural strengthening of RC beams: Behaviour at high temperatures. Constr. Build. Mater..

[4] Tetta Z.C., Bournas D.A. (2016). TRM vs. FRP jacketing in shear strengthening of concrete members subjected to high temperatures. Compos. B. Eng..

[5] Michels J., Zwicky D., Scherer J., Harmanci Y.E., Motavalli M. (2014). Structural Strengthening of Concrete with Fiber Reinforced Cementitious Matrix (FRCM) at Ambient and Elevated Temperature—Recent Investigations in Switzerland. Adv. Struct. Eng..

[6] (1977). DIN 4102–2.

[7] Cerniauskas G., Tetta Z., Bournas D.A., Bisby L.A. (2020). Concrete confinement with TRM versus FRP jackets at elevated temperatures. Mater. Struct..

[8] Ombres L., Mazzuca P., Verre S. (2022). Effects of Thermal Conditioning at High Temperatures on the Response of Concrete Elements Confined with a PBO-FRCM Composite System. J. Mater. Civ. Eng..

[9] Triantafillou T.C., Karlos K., Kefalou K., Argyropoulou E. (2017). An innovative structural and energy retrofitting system for URM walls using textile reinforced mortars combined with thermal insulation: Mechanical and fire behavior. Constr. Build. Mater..

[10] Maroudas S.R., Papanicolaou C.G. (2017). Effect of High Temperatures on the TRM-to-Masonry Bond. Key Eng. Mater..

[11] Ombres L., Iorfida A., Mazzuca S., Verre S. (2018). Bond analysis of the thermally conditioned FRCM-masonry joints. Measurement.

[12] Askouni P.D., Papanicolaou C.C.G., Kaffetzakis M.I. (2019). The Effect of Elevated Temperatures on the TRM-to-Masonry Bond: Comparison of Normal Weight and Lightweight Matrices. Appl. Sci..

[13] Donnini J., De Caso y Basalo F., Corinaldesi V., Lancioni G., Nanni A. (2017). Fabric-reinforced cementitious matrix behavior at high-temperature: Experimental and numerical results. Compos. B Eng..

[14] Ombres L. (2015). Analysis of the bond between Fabric Reinforced Cementitious Mortar (FRCM) strengthening systems and concrete. Compos. B Eng..

[15] Raoof S.M., Bournas D.A. (2017). Bond between TRM versus FRP composites and concrete at high temperatures. Compos. B Eng..

[16] Papanicolaou C.G., Triantafillou T. (2021). Performance of TRM/TRC systems under elevated temperatures and fire conditions. ACI Spec. Publ..

[17] Askouni P.D., Papanicolaou C.G. (2019). Textile Reinforced Mortar-to-masonry bond: Experimental investigation of bond-critical parameters. Constr. Build. Mater..

[18] Carozzi F.G., Arboleda D., Poggi C., Nanni A. (2020). Direct Shear Bond Tests of Fabric-Reinforced Cementitious Matrix Materials. J. Compos. Constr..

[19] De Felice G., D’Antino T., De Santis S., Meriggi P., Roscini F. (2020). Lessons Learned on the Tensile and Bond Behavior of Fabric Reinforced Cementitious Matrix (FRCM) Composites. Front. Built. Environ..

[20] Trochoutsou N., Di Benedetti M., Pilakoutas K., Guadagninia M. (2021). Bond of Flax Textile-Reinforced Mortars to Masonry. Constr. Build. Mater..

[21] D’Antino T., Sneed L.H., Carloni C., Pellegrino C. (2015). Influence of the substrate characteristics on the bond behavior of PBO FRCM-concrete joints. Constr. Build. Mater..

[22] D’Antino T., Gonzalez-Libreros J., Pellegrino C., Carloni C., Sneed L.H., Mechtcherine V., Slowik V., Kabele P. (2018). Performance of Different Types of FRCM Composites Applied to a Concrete Substrate. Strain-Hardening Cement-Based Composites, SHCC 2017.

[23] Raoof S.M., Koutas L.N., Bournas D.A. (2016). Bond between textile-reinforced mortar (TRM) and concrete substrates: Experimental investigation. Compos. B. Eng..

[24] Younis A., Ebead U. (2018). Bond characteristics of different FRCM systems. Constr. Build. Mater..

[25] Tamburini S., Natali M., Garbin E., Panizza M., Favaro M., Valluzzi M.R. (2017). Geopolymer matrix for fibre reinforced composites aimed at strengthening masonry structures. Constr. Build. Mater..

[26] Li T., Zhang Y., Dai J.G. (2017). Flexural behavior and microstructure of hybrid basalt textile and steel fiber reinforced alkali-activated slag panels exposed to elevated temperatures. Constr. Build. Mater..

[27] Longo F., Lassandro P., Moshiri A., Phatak T., Aiello M.A., Krakowiak K.J. (2020). Lightweight geopolymer-based mortars for the structural and energy retrofit of buildings. Energy Build..

[28] (2005). CEN. EN 1996-1-1 Eurocode 6–Design of Masonry Structures–Part 1-1: General Rules for Reinforced and Unreinforced Masonry Structures.

[29] (1991). RILEM TC 76: Technical Recommendations for Testing and Use of Constructions Materials: LUMB1-Compressive Strength of Small Walls and Prisms.

[30] (1999). CEN. EN ISO 13934-1: Textiles-Tensile Properties of Fabrics–Part 1: Determination of Maximum Force and Elongation at Maximum Force Using the Strip Method.

[31] (1993). CEN. EN 1015-11: Methods of Test for Mortar for Masonry–Part 11: Determination of Flexural and Compressive Strength of Hardened Mortar.

[32] (2016). AC434 ICC-ES: Masonry and Concrete Strengthening Using Fiber-Reinforced Cementitious Matrix (FRCM) Composite Systems.

[33] De Felice G., Aiello M.A., Caggegi C., Ceroni F., De Santis S., Garbin E., Gattesco N., Hojdys Ł., Krajewski P., Kwiecień A. (2018). Recommendation of RILEM Technical Committee 250-CSM: Test method for Textile Reinforced Mortar to substrate bond characterization. Mater. Struct..

